# Full mouth functional and aesthetic rehabilitation of a patient affected with hypoplastic type of amelogenesis imperfecta

**DOI:** 10.4317/jced.56217

**Published:** 2020-03-01

**Authors:** Muhammad-Rizwan Nazeer, Robia Ghafoor, Kamil Zafar, Farhan-Raza Khan

**Affiliations:** 1 BDS, FCPS, Senior registrar in Operative Dentistry at the Baharia University Medical and Dental College, Karachi, Pakistan; 2 BDS, FCPS, Consultant in Operative Dentistry at the Aga Khan University Hospital, Karachi, Pakistan; 3 BDS, Instructor in Operative Dentistry at the Aga Khan University Hospital, Karachi, Pakistan; 4 BDS, MCPS, FCPS, and M.Sc., Consultant in Operative Dentistry at the Aga Khan University Hospital, Karachi, Pakistan

## Abstract

The management of Amelogenesis imperfecta often poses a challenge for the dentists. It not only includes aesthetic and functional rehabilitation of the patient, but also requires a positive rapport building with the patient due to psychosocial issues. The treatment plan is driven by patient demands, age, cost-affordability, severity of the disease and the presenting condition. The present case report elucidates step by step management of a 20 year-old female who presented with generalized hypersensitivity, intermittent pain associated with multiple decayed posterior teeth, poor dental aesthetics and anterior deep bite. The management consisted of endodontic treatments in all teeth, crown lengthening to gain ferrule in some teeth, provision of provisional bridges at an increased vertical dimension for six weeks followed by full mouth all ceramic crowns on all teeth. The prosthetic management aimed at reorganized occlusal scheme. There was a significant improvement in the aesthetics, deep bite, and along with correction of the vertical dimension of occlusion.

** Key words:**Amelogenesis imperfecta, hypoplastic enamel, mouth rehabilitation, dental esthetics.

## Introduction

Amelogenesis imperfecta (AI) is the most common inherited disorder that cause abnormalities in composition and morphology of enamel (1). Its prevalence studied among different population groups varies between 1:700 to 1:16000 (2). The mode of inheritance of Amelogenesis Imperfecta can be X-linked, autosomal recessive and / or autosomal dominant (3). It affects both deciduous and permanent dentitions (1). It is caused by mutation in the genes that encodes information for enamel phenotype, the common being amelogenin (4). Amelogenesis imperfecta cane be further classified into four main types, which are Hypoplastic, Hypoclacified, Hypomaturation and Hypomaturation- Hypoplastic with Taurodontism (5). Hypoplastic AI is characterized by diffuse pitting or grooves caused by defective matrix deposition resulting in reduced enamel thickness. Hypocalcified AI occurs due to deficient enamel calcification. The affected enamel appears chalky white, with normal enamel thickness. Tooth structure is weak which rapidly wears off resulting in dentine exposure. In Hypomaturation type, overall enamel thickness is normal but with mottled look. In Hypomaturation- Hypoplastic with Taurodontism type the appearance is mottled with diffuse pitting. The pulp chamber is large with furcation located more apically. The clinical presentation of affected individuals are highly variable (1). There are multiple dental issues associated with Amelogenesis imperfecta (AI). It includes sensitivity, surface roughness, and discoloration and/ or may result in loss of vertical dimension due rapid tooth wear. Such issues may pose serious aesthetic, functional and psychological concerns to the patient (5,6). The management of Amelogenesis imperfecta often pose a challenge for clinicians. It not only includes aesthetic and functional rehabilitation of the patient, but also requires a positive rapport building with the patient due to psychosocial withdrawal. The treatment plan is driven by patient demands, age, financial status, severity of the disease and the presenting condition to a clinician (7). The current case report elucidates step by step management of a young lady who presented with generalized hypersensitivity, intermittent pain and poor aesthetics.

## Case Report

A 20 year-old female presented to the dental clinics with the complaint of unaesthetic smile, generalized sensitivity, difficulty in chewing due to multiple cavities for several months and severe intermittent pain in left upper tooth for 5 days. The pain was spontaneous; aggravated on taking hot and cold fluids and during chewing. Pain was relieved temporarily after taking analgesics. Due to broken down posterior teeth and generalized sensitivity, she experienced extreme discomfort during mastication of hard or fibrous food, therefore, she relied more on soft, refined food for several years and used to skips regular meals. Most of her meals consisted of sweet drinks and refined carbohydrates. Her mother informed that she had suffered same problems with the deciduous dentition. Family history revealed that her grandfather had the same dental problem and most of his teeth were removed in early age. The oral hygiene history was also not satisfactory. The patient was otherwise healthy with no known allergies or co-morbids. She visited multiple dentists previously but was not satisfied with the given treatment plans. Most of them advised her extraction of most of her posterior teeth but she was willing to save her natural teeth. She was well motivated towards dental treatment and had no financial constraints. Therefore, wanted a durable solution with perfect aesthetics at the same time requested retaining her natural teeth. 

**Figure 1:**
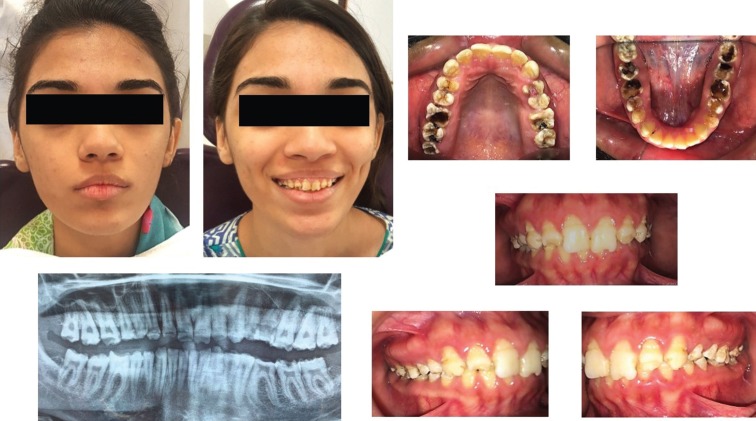
Pre-operative clinical pictures and panoramic radiograph.

Extra oral examination revealed normal mouth opening with no tenderness, clicking or crepitus over the Temporo-mandibular joint. The lower facial height was reduced. Intraoral examination revealed poor oral hygiene with grossly carious posterior teeth. The occlusal plane was uneven with deep anterior bite and worn-down posterior teeth. The labial surfaces of all anterior teeth were stained due to irregular grooving. The overall enamel thickness was also reduced. The smile line was average but non-consonant. Marginal gingiva was red, inflamed and edematous. Deep periodontal pockets of 4mm were found around left mandibular first and second molar, and right mandibular first molar, however 2.5- 3mm pocket depth was found in all other posterior teeth. Tooth wear was uncompensated with freeway space of approximately 4-5mm.The panoramic radiograph revealed substantial loss of enamel from the occlusal surfaces of all teeth with multiple decayed lesions. Preoperative clinical pictures and panoramic radiograph are shown in Figure 1. Diagnostic casts were obtained, on which occlusion was evaluated. On the basis of clinical and radiographical findings, the most probable diagnosis was of hypoplastic type Amelogenesis imperfecta. The problem list consisted of:

1. Poor oral hygiene with generalized gingivitis and localized periodontitis.

2. Multiple carious lesions involving all posterior teeth.

3. Generalized attrition with loss of occlusal vertical dimensional dimension.

4. Compromised esthetics.

The aim of treatment was to provide a functional occlusion with realistic aesthetics and prevention of further tooth loss. The patient was asked to maintain a regular food diary to found out the frequency of cariogenic diet along with record of oral hygiene aids. This helped us in changing her diet to healthier and more fibrous diet. Mock build-up of full coverage crowns were initially done on upper and lower casts and showed to the patient. She was satisfied with the predicTable outcome. So the following treatment plan was made: 

Immediate management: Endodontic treatment of tooth #24 for relieving patient pain 

Definitive treatment plan:

1. Oral hygiene instructions, patient motivation and dietary modifications.

2. Endodontic treatments of all natural teeth excluding of tooth #24 and third molars (#18, 28, 38, 48).

3. Extraction of all third molars (#18, 28, 38, 48).

4. Crown lengthening surgery on tooth #36, 37 and #46 for gaining ferrule.

5. Restoration of the lost occlusal vertical dimension by provision of temporary bridges on all posterior teeth and freehand composite buildup on all palatal surfaces of upper teeth and lower canines. 6. Bisque trial and provision of all ceramic crowns.

7. Provision of a Michigan type stabilization splint.

8. Reinforcement of oral hygiene measures and periodic follow-ups.

**Figure 2:**
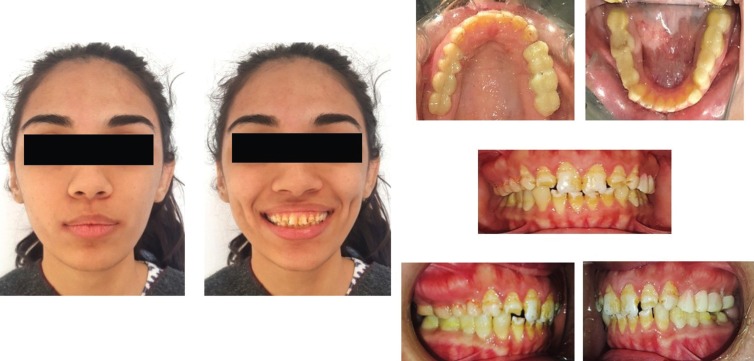
Temporary acrylic bridges on all posterior teeth and composite on palatal surfaces of upper anterior and lower canine teeth.

**Figure 3:**
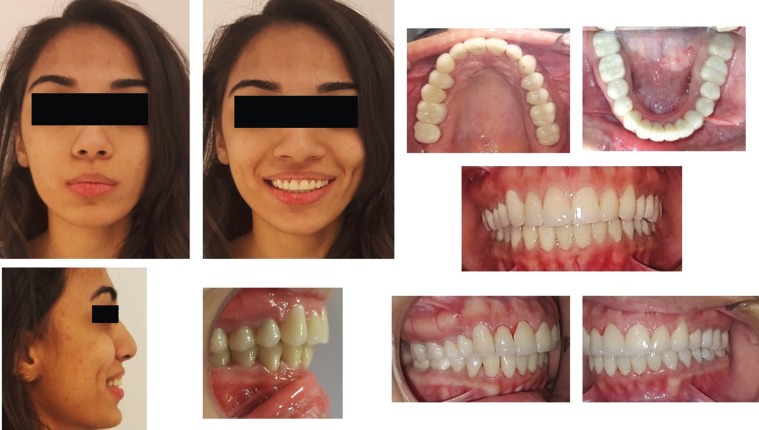
Post-operative extra-oral and intraoral pictures.

**Figure 4:**
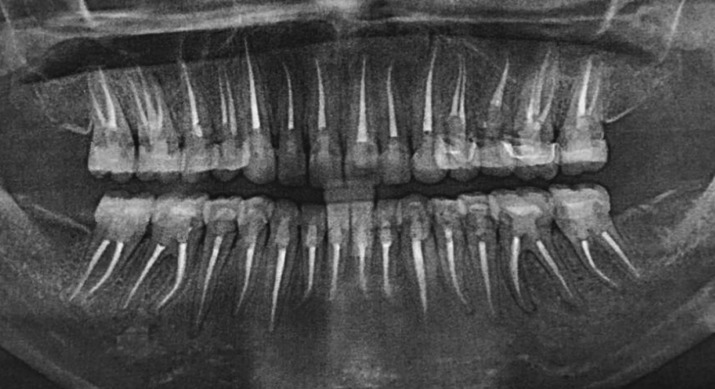
Postoperative panoramic view.

**Figure 5:**
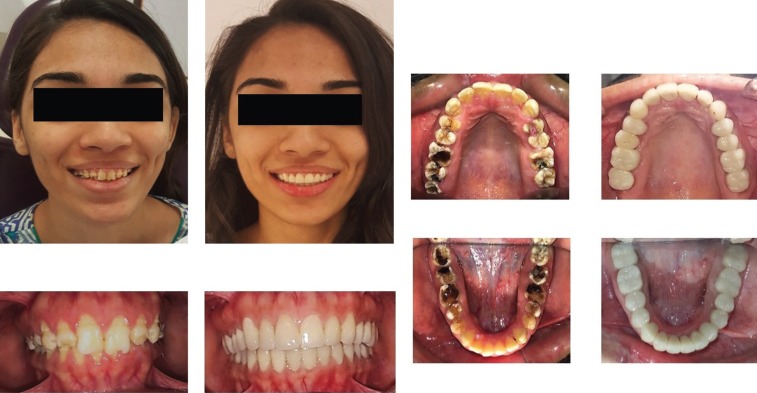
Pre and post-operative comparative clinical pictures.

Initially, endodontic treatment was performed on #24 to relief patient symptoms, then it was done on all remaining natural teeth and post endodontic build-up was done with the composite ( 3M™ ESPE™ Filtek™ P60 Posterior Restorative System). All wisdom teeth were extracted and gingivectomy was performed on #36, 37 and #46 to gain ferrule for subsequent crown placement. After healing, diagnostic impressions were made using irreversible hydrocolloid (Tropicalgin, Zhermack Italy). Diagnostic casts were mounted on semi adjusTable articulator (Hanau Articulator, Teledyne Hanau Buffalo, NY, USA) using Hanau facebow. Diagnostic wax up was done according to optimum aesthetics and function followed by fabrication of a vacuum form stent (3A MEDES, EASY – VAC GASKET, KOREA). All posterior teeth were prepared and temporary acrylic bridges (Integrity™ Temporary Crown & Bridge Material, DENTSPLY, USA) were placed at an increased occlusal height. Composite buildups (3M™ ESPE™ Filtek™ P60 Posterior Restorative System, USA) on all palatal surfaces of upper teeth and lower canines as shown in Figure 2. Temporary bridges were left for three months to evaluate patient’s tolerance to increase occlusal vertical dimension (OVD). Periodic follow-ups were done after a week, one month and three months to assess the patients tolerance and any associated complication of temporary bridges. Patient was comforTable, no muscle tension was noted and TMJ examination revealed no tenderness or crepitus. Therefore, definitive impressions of maxillary and mandibular arch were taken with poly vinyl polysiloxane impression material (Aquasil Ultra Putty Soft Regular and Aquasil Ultra LV, Dentsply USA). Interocclusal records were taken in centric relation using bimanual manipulation method. Working casts were then articulated using interocclusal records on semi-adjusTable articulator (Hanau Articulator, Teledyne Hanau Buffalo, NY, USA). A mutually protected occlusal scheme was preserved to avoid stresses on posterior teeth during lateral excursions. All ceramic crowns (IPS e-max Ceram, Ivoclar vivadent, Leichtenstein. Germany) were fabricated and bisque bake trial was done for the evaluation of crown contours, contacts, occlusion and aesthetics. Crowns were glazed and bonded to the teeth with self-adhesive resin (RelyX Unicem 2 Clicker A2 Universal Self-Adhesive Universal Resin, 3M-ESPE, USA). A Stabilization splint was made after the delivery of final prosthesis and delivered to the patient. Post-operative extra-oral and intraoral pictures shown in Figure 3 displays a significant improvement in esthetics and even occlusal plane with correction and increase in vertical dimension of occlusion. Postoperative panoramic view is shown in Figure 4 displays nicely done endodontics and fixed prosthodontics. Pre and post-operative comparative clinical pictures are shown in Figure 5 showing significant improvement in dento-facial aesthetics. Patient was asked to follow up in six months.

## Discussion

In hypoplastic type of AI, the affected enamel has defective enamel matrix proteins resulting in altered morphologic features of dental enamel (8). In our patient, hypoplastic form predominates, because irregular grooving was found on the facial and buccal surfaces of all teeth affecting the overall thickness of enamel and staining more pronounced on anterior teeth. Our patient had poor oral hygiene because most of her diet consisted of refined carbohydrates further aggravating the condition. Poor oral hygiene and atypical morphology of tooth due to hypoplastic enamel are the most probable reasons that all posterior teeth were carious. Amelogenesis imperfecta is also associated with certain skeletal and dental defects such as skeletal open bite, constricted maxillary arch, reverse curve of spee and vertical growth pattern etc (3). Constricted maxillary arch with uneven occlusal plane and vertical growth pattern was also observed in our patient, however we found deep bite instead of open bite. Rapid wearing of posterior teeth due to developmental defect and subsequent dental caries are the most probable reason for deep anterior bite and uneven occlusal plane. The management of amelogenesis imperfecta is always challenging and required multidisciplinary approach. Depending on the severity of disease and patient demands, various treatment options can be suggested which includes direct restorations, indirect restorations which includes veneers or full coverage restorations and sometimes even extraction of teeth followed by fixed prosthodontics, implant retained restorations or removable prosthodontics (9,10). The enamel was deficient and malformed, resulted in poor esthetics, staining, caries leading to tooth sensitivity as experienced by the patient. Therefore, the best treatment plan keeping in mind the patient demands, financial status, clinical and radio graphical findings, consisted of salvaging all natural teeth, doing crown lengthening surgeries in areas with inadequate ferrule followed by provision of full coverage crowns on all teeth. Most of her posterior teeth were carious with pulpal exposure, therefore endodontic treatment was performed on all of her posterior teeth before going for conventional fixed prosthodontics. For anterior teeth initial plan was to give indirect veneers for masking unaesthetic appearance and for improving shape and colour. Due to dentin exposure in lower anterior teeth, deep grooving in upper anterior teeth, extremely deficient enamel required for bonding of veneers seems unpredicTable solution for her teeth. Marginal adaptation and post-operative sensitivity were the other issues related to veneers placement, therefore full coverage restorations were also planned for anterior teeth. The risk of endodontic complications that is loss of vitality, pulpitis, and apical periodontitis after crowns in amelogenesis imperfecta were reported to be 3-7.6% (11,12). Although the risk was low, but considering the young age and severity of the disease and to avoid any complications following tooth preparation, endodontic treatment of all anterior teeth were electively done. A similar sort of cases were also reported in the literature in which due to severity of the disease all teeth were endodontically treated before prosthetic rehabilitation (13-15).

With modifications in various types of ceramic in dentistry, all ceramic crowns now provides a durable and appealing results when placed on anterior or posterior tooth (16-19). IPS e.max is lithium disilicate ceramic which provides best combination of aesthetics, fit, strength and bonding to the natural tooth structure. It requires less tooth reduction as compare to metal ceramic or zirconia based restorations, so more natural tooth is conserved (7). The post endodontic buildup in all posterior teeth comprised mainly of composite restoration, therefore by placing all ceramic crowns a reliable bonding could be achieved with both the composite restorations and the remaining natural tooth structure. All ceramic crowns were also preferred by various other authors in managing patients with amelogenesis imperfect (7,17-19).

Loss of OVD is also a common presentation in patients suffering from amelogenesis imperfect (11). It results in an uneven occlusal plane resulting in development of occlusal interferences, further aggravating the condition. The treatment plan consisted of regaining the lost OVD together with provision of even occlusal plane for optimum function and esthetics. One of the method for increasing OVD by providing temporary bridges at an increased OVD for minimum 4-6 weeks. This was done to accustom temporomandibular joint to the new OVD dimension and to prevent technical complication in newly placed indirect restoration (20,21). Our patient had moderate loss of occlusal vertical dimension (OVD that was uncompensated with freeway space of 4-5mm along with anterior deep bite. So we kept the patient provisional acrylic bridges for a period 6 weeks before placement of definitive fixed restorations. Along with the correction of occlusal vertical dimension, deep anterior bite was also significantly improved.

Dental smile has an important psycho-social impact on human personality. The unaesthetic smile display in patients suffering from amelogenesis imperfecta results in social withdrawal and development of psychological problems (22). To prevent these happenings restorations with best esthetic should be planned. Therefore we planned all ceramic crowns which provided the best combination of aesthetics and protection of remaining natural tooth structure. The final smile, colour, shape, contours and marginal fit of all ceramic crowns were excellent. The maxillary arch in our patient was constricted, therefore after placement of final crowns buccal fullness was achieved which masked the vertical growth pattern of our patient. Dentofacial aesthetics ware significantly improved along with masticatory function. Patient’s chewing habit was significantly following treatment as she could eat hard or fibrous food without any discomfort therefore was extremely happy and satisfied with the outcome of treatment.

Maintaining oral hygiene is teeth with defective enamel because the irregular tooth surfaces are more prone to plaque retention. We placed separate crowns on all teeth with anatomical contacts so that oral hygiene can be easily maintained. Separated crowns were also preferred by various authors (7,8,10,15,19,23). However, these patients should be kept of regular follow-ups and meticulous oral hygiene measures should be reinforced.

## Conclusions

The presented case report explains step by step multidisciplinary management of a 20 year-old female who presented with Amelogenesis imperfecta. Multidisciplinary approach comprises of coordinated restorative, prosthodontics and periodontics procedures at an increased vertical dimension of occlusion. Patients with hypoplastic defects, presenting with moderate to severe decay and diffuse grooving of anterior teeth, all ceramic crowns lithium disilicate crowns are the most conservative, durable and predictable treatment option because of their excellent aesthetics, durability, strength, marginal fit and biocompatibility.

## Clinical relevance

Patients with hypoplastic defects, presenting with moderate to severe decay and diffuse grooving of anterior teeth, all ceramic crowns lithium disilicate crowns are the most conservative, durable and predicTable treatment option because of their excellent aesthetics, durability, strength, marginal fit and biocompatibility. This article aims to illustrate the planning and restorative management of a patient who presented with Amelogenesis Imperfecta.
